# Curcumin-Added Whey Protein Positively Modulates Skeletal Muscle Inflammation and Oxidative Damage after Exhaustive Exercise

**DOI:** 10.3390/nu14224905

**Published:** 2022-11-19

**Authors:** Kelly Aparecida Dias, Aline Rosignoli da Conceição, Stephanie Michelin Santana Pereira, Lívya Alves Oliveira, Jojo Silva Rodrigues, Roberto Sousa Dias, Sérgio Oliveira de Paula, Antônio José Natali, Sérgio Luis Pinto da Matta, Reggiani Vilela Gonçalves, Elad Tako, Hercia Stampini Duarte Martino, Ceres Mattos Della Lucia

**Affiliations:** 1 Department of Nutrition and Health, Universidade Federal de Viçosa, Viçosa 36570-900, MG, Brazil; kelly.dias@ufv.br (K.A.D.); aline.conceicao@ufv.br (A.R.d.C.); stephanie.pereira@ufv.br (S.M.S.P.); livya.oliveira@ufv.br (L.A.O.); hercia72@gmail.com (H.S.D.M.); 2 Department of General Biology, Universidade Federal de Viçosa, Viçosa 36570-900, MG, Brazil; jorodriguez6694@gmail.com (J.S.R.); rosousa318@gmail.com (R.S.D.); depaula@ufv.br (S.O.d.P.); smatta@ufv.br (S.L.P.d.M.); 3 Department of Physical Education, Universidade Federal de Viçosa, Viçosa 36570-900, MG, Brazil; anatali@ufv.br; 4 Department of Animal Biology, Universidade Federal de Viçosa, Viçosa 36570-900, MG, Brazil; reggiani.goncalves@ufv.br; 5 Department of Food Science, Cornell University, Stocking Hall, Ithaca, NY 14850, USA; et79@cornell.edu

**Keywords:** exhaustive swimming test, curcuma, bioactive compounds, antioxidants, inflammatory process, oxidative stress, experimental study

## Abstract

(1) Background: Exhaustive exercise can induce muscle damage. The consumption of nutritional compounds with the ability to positively influence the oxidative balance and an exacerbated inflammatory process has been previously studied. However, little is known about the nutritional value of curcumin (CCM) when mixed with whey protein concentrate (WPC). This study was developed to evaluate the effect of CCM-added WPC on inflammatory and oxidative process control and histopathological consequences in muscle tissue submitted to an exhaustive swimming test (ET). (2) Methods: 48 animals were randomly allocated to six groups (*n* = 8). An ET was performed 4 weeks after the start of the diet and animals were euthanized 24 h post ET. (3) Results: WPC + CCM and CCM groups reduced IL-6 and increased IL-10 expression in muscle tissue. CCM reduced carbonyl protein after ET compared to standard AIN-93M ET and WPC + CCM ET diets. Higher nitric oxide concentrations were observed in animals that consumed WPC + CCM and CCM. Consumption of WPC + CCM or isolated CCM reduced areas of inflammatory infiltrate and fibrotic tissue in the muscle. (4) Conclusions: WPC + CCM and isolated CCM contribute to the reduction in inflammation and oxidative damage caused by the exhaustive swimming test.

## 1. Introduction

Exhaustive exercise can induce muscle damage [1,2], which, consequently, causes the development of inflammatory responses and the production of reactive oxygen species (ROS) and free radicals inducing oxidative stress in the muscle tissue [2]. Inflammation and oxidative stress induced by exhaustive exercise are indispensable for tissue recovery; however, when exacerbated and uncontrolled, they can cause secondary muscle damage [1,3]. Thus, strenuous exercise and secondary muscle damage can reduce muscle strength, impaired neuromuscular function, fatigue, a reduced range of motion, and muscle soreness [4,5,6]. In addition, this muscle damage can decrease muscle function, affecting physical activities and performance [3,7]. Therefore, strategies that control or minimize muscle damage, attenuate exacerbated inflammatory responses, and improve performance are increasingly studied [6,8].

In this context, consuming products with antioxidant and anti-inflammatory properties has become common and necessary to treat muscle damage [4,8]. Among the nutritional compounds that are capable of promoting a good response in the function muscle tissue is curcumin (CCM), the main curcuminoid extracted from *Curcuma longa* L, which has received considerable attention. Studies have shown that CCM can positively affect different illnesses due to its antioxidant, anti-inflammatory, and immunoregulatory effects, which highlight its cardioprotective, antineoplastic, and hepatoprotective potential [9,10,11]. In addition to its demonstrated health benefits, polyphenol curcumin has also been shown to improve performance in physical exercise by reducing muscle pain and damage [3,12,13,14], which is characterized by sarcolemma disruption, extracellular myofiber matrix abnormalities, swelling or disruption of the sarcotubular system, and distortion of the myofibril contractile components, to name a few [15].

Whey proteins (WP) have become widely consumed as a nutritional supplement because of their leucine content, digestibility, and ability to activate muscle protein synthesis [11,16]. In addition to their efficacy and safety, these proteins have high biological value and metabolic efficiency, optimizing recovery from strenuous exercise and sports performance, strength, and muscle hypertrophy [17,18]. Studies suggest that the use of WP can also attenuate oxidative stress and exercise-induced inflammation, as well as aid recovery after resistance training [16,19,20]. The benefits of WP are mainly related to its concentration of bioactive peptides [21]. In addition, it was previously demonstrated that WP rich in leucine acts as an anabolic signal, decreasing the levels of ubiquitinated proteins and inhibiting proteasome activity, decreasing muscle atrophy [22].

Previous studies have shown that CCM and whey protein ingestion alone can attenuate muscle damage induced by unusual exercise [3,18,20,23,24]. However, current scientific knowledge is lacking in regard to the potentiation of the beneficial effects of CCM-added WP, especially concerning the maintenance of muscle tissue integrity, the improvement of the oxidative balance, or the attenuation of inflammatory responses, and consequently, the histopathological changes in the muscle during the practice of strenuous exercise. Thus, and in order to bridge this gap, the aim of the present study was to evaluate the effects of curcumin-added whey protein concentrate (WPC) on the oxidative balance, inflammation process, and histopathology changes of the muscle of Wistar rats submitted to an acute protocol of exhaustive physical exercise.

## 2. Materials and Methods

### 2.1. Feedstock

#### 2.1.1. Curcumin

The curcumin used in this study was Theracurmin^®^ (Theravalues, Tokyo, Japan), which is highly absorbable. This product has more bioavailability than conventional CCM.

#### 2.1.2. Whey Protein Concentrate (WPC)

The milk for the manufacture of whey protein concentrate was obtained from the stable of the Federal University of Viçosa-MG, being submitted to microfiltration. The whey-drying procedure was carried out in a LabMaq MSD 1.0 single-level spray dryer (Ribeirão Preto, SP, Brazil), according to the methodology of Perrone et al. (2013) [25].

#### 2.1.3. Curcumin-Added Whey Protein Concentrate

CCM was mixture to WPC to obtain a product with a concentration of 0.8 g of curcumin in every 100 g of WPC, and an intake of 3.0 mg/kg/day of curcumin (maximum ADI value for humans [26]) was considered for a 70 kg individual [27]. Therefore, the protein supply to the animals through WPC corresponded to 30% of the requirements for rats in the maintenance phase [28].

### 2.2. Animals and Experimental Diets

**Figure 1:**
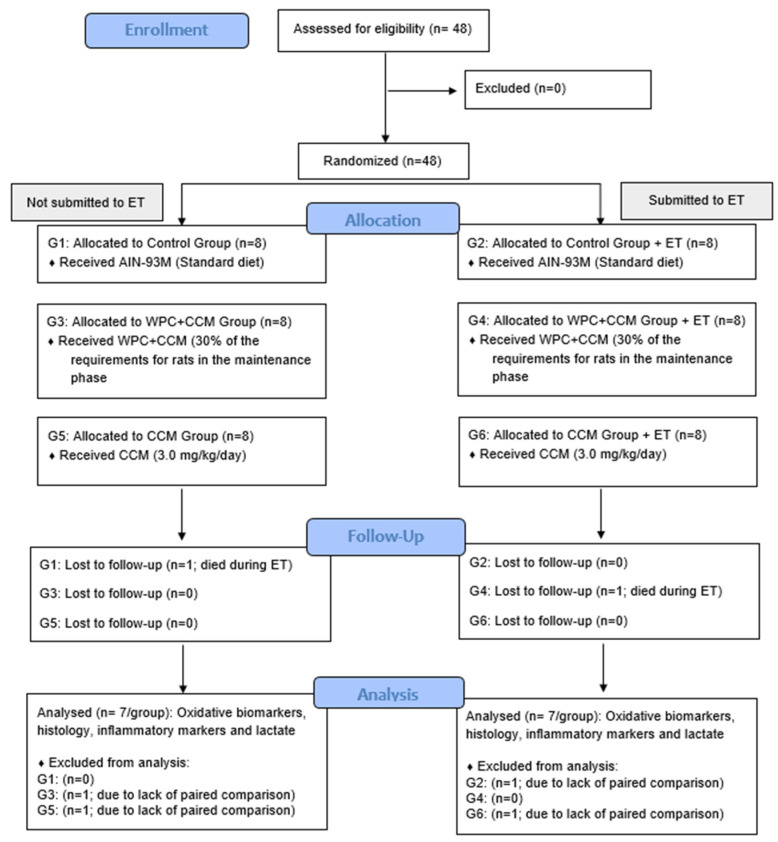
Flow diagram depicting the study design. AIN-93M: group that received a standard diet; WPC + CCM ET: group that received curcumin-added whey protein concentrate; CCM: group that received curcumin; ET: exhaustion test. Based on CONSORT [28].

Forty-eight male rats (young adults) of the Wistar strain (Rattus novergicus, variety albinus, Rodentia), 12 weeks old, were randomly divided into six experimental groups with 8 animals each (*n* = 8/group), as follows: (i) the control group that received a standard diet (AIN-93M); (ii) the control group that received a standard diet submitted to the exhaustive test (AIN-93M ET); (iii) the curcumin-added whey protein concentrate group (WPC + CCM); (iv) the curcumin-added whey protein concentrate group submitted to the exhaustive test (WPC + CCM ET); (v) the curcumin group (CCM); and (vi) the curcumin group submitted to the exhaustive test (CCM ET) (Figure 1). 

The experimental diets specific to each group (formulated and produced in the laboratory of experimental nutrition at UFV, according to the AIN-93M recommendations proposed by the American Institute of Nutrition (1993) [29]) were introduced and maintained for 4 weeks (Table 1). The animals received water and their experimental diets ad libitum. Diets were normocaloric, normoproteic and normolipidic. The animals were distributed in individual cages in a temperature-controlled environment (22 °C ± 2 °C) and automatically controlled 12 h light/dark cycles.

The animals from the groups AIN-93M ET, WPC + CCM ET, and CCM ET were submitted to the exhaustive test after 4 weeks of experiment. The rats swam to exhaustion, supporting a loading equivalent to 4% of the body weight fixed to the tail (the exhaustion protocol was modified) [30]. Exhaustion was defined as when the animal remained submerged for 10 s [31].

At the end of the experimental period and 24 h after the exhaustive test, the animals were anesthetized with isoflurane (Isoforine, Cristália^®^, Itapira, Brazil) and euthanized. Skeletal muscle tissues (gastrocnemius and biceps) were removed, weighed, frozen in liquid nitrogen, and stored at −80 °C until analysis. All experimental procedures were carried out following the ethical principles of animal experimentation.

### 2.3. Analysis of Oxidation Products and Activity of Antioxidant Enzymes

The muscle tissue samples (gastrocnemius) were weighed (300 mg) and homogenized in 1.5 mL of cold PBS (pH 7.4) using an Ultra-Turrax homogenizer (T10 basic Ultra-Turrax, IKA^®^, Campinas, SP, Brazil). The homogenate was centrifuged at 10,000× *g* for 10 min at 4 °C. The supernatant was used for enzymatic analysis and the evaluation of oxidation biomarkers.

Catalase (CAT), determined by its ability to cleave hydrogen peroxide (H_2_O_2_) into water and molecular oxygen, was evaluated as proposed by Aebi (1984) [32]. Superoxide dismutase (SOD) activity, defined as the amount of enzyme that causes a reduction in pyrogallol autooxidation, was performed according to the method proposed by Dieterich (2000) [33]. The activity of glutathione-S-transferase (GST) was obtained from its ability to metabolize 1-chloro-2,4-dinitrobenzene (CDNB), conjugated to reduced glutathione, following the methodology described by Habig (1981) [34].

The concentration of malondialdehyde (MDA) was determined as thiobarbituric acid reactive substances (TBARS) of lipid peroxidation, according to the methodology proposed by Buege and Aust (1978) [35]. The production of NO was estimated by the production of NO_2_/NO_3_ by the standard Griess reaction and followed the methodology of TSIKAS (2007) [36], and the oxidative damage of muscle proteins, indicated by the levels of carbonyl proteins (CP), was measured according with the method of Levine (1990) [37]. The results were normalized to the total protein concentration of the supernatant using the method described by Lowry (1951) [38].

### 2.4. Cytokine Profile in Muscle Homogenate

The muscle samples (200 mg) were homogenized using a tissue homogenizer (IKA WORKS GMBH and CO, Staufen, BW, Germany, model T10) in PBS buffer (pH 7.0), centrifuged (10,000× *g*, for 10 min at 4 °C), and the supernatant recovered. The cytokines Interleukin-2, Interleukin-10, and tumor necrosis factor were determined by a FACSVerse flow cytometer (BD, Franklin Lakes, NJ, USA) by the Cytometric Bead Array (CBA) following the manufacturer’s recommendations (BD). Data were processed using FCAP Array v3.0 software, and the results were expressed in pg/g tissue.

### 2.5. Real-Time Polymerase Chain Reaction (RT-qPCR)

The relative expression level was assessed by a qPCR in an Illumina Eco^®^ real-time polymerase chain reaction system (Illumina, San Diego, CA, USA), using the GoTaq^®^ 1-Step RT-qPCR System (Promega, Madison, WI, USA). After extraction, the RNA from the muscle samples (gastrocnemius) was quantified using the Qubit 4 Fluorometer (Thermo Scientific). The sequences of the primers that were used are described in Table 2. The analysis was performed using the 2−ΔCt method in EcoStudy^®^ software (Illumina, San Diego, CA, USA), using GAPDH as an endogenous control.

### 2.6. Histopathological Analyzes

The muscle samples (biceps) were removed and fixed in formaldehyde. After fixation, the tissues were dehydrated in ethanol and embedded in resin containing Historesin^®^ hardener (Leica, Wetzlar, HE, Germany). Histological sections were obtained using an automatic microtome (Leica). They were then submitted to staining using the hematoxylin and eosin (H&E) technique, mounted with Entellan (Merck), and analyzed under a light microscope (Leica DM750).

The images of the histological sections were obtained with a 20× objective using the LEICA MC170 HD digital camera. From 10 photos per histology slide, the areas containing muscle fiber, connective tissue (which was considered fibrosis when the quantity of collagen was exacerbated), and inflammatory infiltrate in the muscle were quantified using the ImagePro-Plus^®^ application version 4.5 (Media Cybernetics, Rockville, MD, USA) by manually counting points on the tissue [39].

### 2.7. Blood Lactate Analysis during the Exhaustion Test

Blood samples (25 μL) were collected at two stages: before (rest) and after the end of the exhaustive swimming test (final 60 s of testing), following the protocol of Gobatto et al. (2001) [40], with modifications. The blood samples were collected from the tail end of the animal (*n* = 8). The animals were quickly dried with a towel before blood collection to avoid dilution with water. Lactate concentration was determined using the Accutrend Check automated blood lactate analyzer (Accusport—Roche, Basel, Switzerland).

### 2.8. Statistical Analysis

The results were expressed as mean and standard deviation (mean ± SD). Data normality was assessed using the Kolmogorov–Smirnov test. The *t*-test evaluated differences within the same group (not submitted to ET × submitted to ET). Intra-group differences (AIN-93M, WPC + CCM, and CCM) were submitted to an analysis of variance (ANOVA), followed by the Newman–Keuls mean test at 5% probability. All statistical analyses and graph construction were performed using the GraphPad Prism software, version 8 (GraphPad Prism Inc, La Jolla, CA, USA).

## 3. Results

### 3.1. Antioxidant Enzymes and Markers Oxidatives

**Figure 2:**
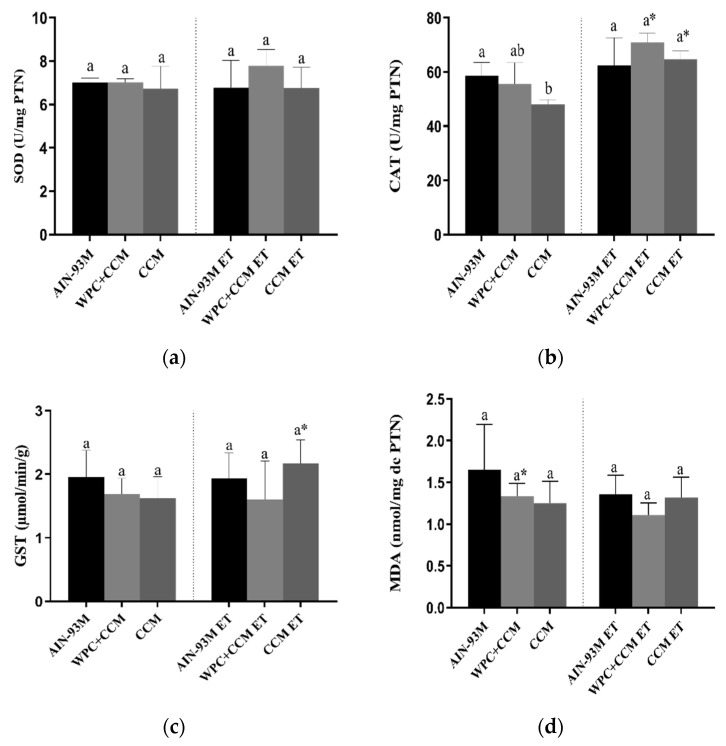

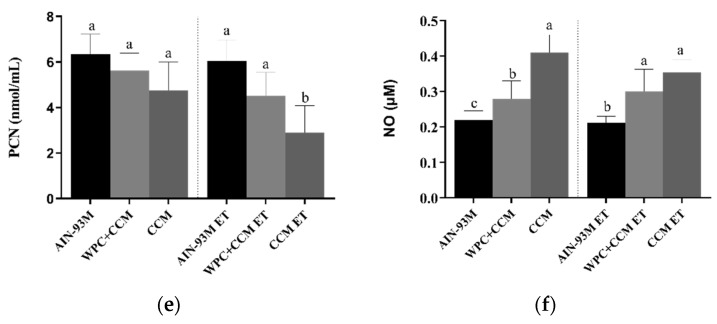
Analysis of oxidative balance in muscle. AIN-93M: group that received a standard diet; AIN-93M ET: group submitted to the exhaustion test that received a standard diet; WPC + CCM: group not submitted to ET that received curcumin-added whey protein concentrate; WPC + CCM ET: group submitted to the exhaustion test that received curcumin-added whey protein concentrate; CCM: group not submitted to ET that received curcumin; CCM ET: group submitted to the exhaustion test that received curcumin; ET: exhaustion test. The graphs show (**a**) superoxide dismutase, (**b**) catalase, (**c**) glutathione S-transferase, (**d**) malondialdehyde, (**e**) carbonylated protein, and (**f**) Nitric Oxide. * indicates significant differences between groups that were not submitted to ET and groups that were submitted to ET, according to the *t*-test (*p* < 0.05). According to ANOVA, different lowercase letters (**a**–**c**) indicate significant mean differences among the groups, followed by the Newman–Keuls test, at 5% probability. Same lowercase letters indicate that there was no significant mean difference among the groups. Data expressed as mean ± standard deviation.

The SOD activity did not differ among the AIN-93M, WPC + CCM, and CCM groups, regardless of whether or not ET was performed (Figure 2a). Among the animals not submitted to ET, the CAT activity was higher in the AIN-93M group when compared to the animals that consumed CCM (*p* < 0.05). The coefficient of variation (CV) among groups showed variation of 2.4 to 19% (Table 3). On the other hand, the CAT activity was not altered in the animals of the groups submitted to ET. The animals in the WPC + CCM ET group, as well as the animals in the CCM ET group, showed higher CAT activity compared to the animals in the WPC + CCM and CCM group, respectively (*p* < 0.05) (Figure 2b). The groups showed a CV range of 3.5 to 15.7% (Table 3). Glutathione activity, in turn, also did not differ among groups, regardless of whether the ET was performed. However, the animals in the CCM ET group showed higher GST activity when compared to the CCM group not submitted to ET (*p* < 0.05) (Figure 2c). The CV range was from 15.6 to 41.5% (Table 3).

MDA concentrations showed no difference among the AIN-93M, WPC + CCM, and CCM groups, regardless of whether ET was performed. However, the animals in the WPC + CCM ET group had lower concentrations of MDA when compared to the WPC + CCM group (*p* < 0.05) not submitted to ET (Figure 2d). The groups showed a CV range of 11.2 to 33.7% (Table 3). PCN concentrations were similar among the animals of the AIN-93M, WPC + CCM, and CCM groups not submitted to ET. However, the animals in the CCM ET group had lower concentrations of PCN (*p* < 0.05) when compared to the animals in the AIN-93M ET group and the WPC + CCM ET group (Figure 2e). The CV among groups showed variation of 13.7 to 42.8% (Table 3). Among animals not submitted to ET, NO levels were higher in the CCM group when compared to the AIN-93M and WPC + CCM groups (*p* < 0.05). Moreover, the WPC + CCM group had a higher amount of NO when compared to the AIN-93M group (*p* < 0.05). Concerning the animals submitted to ET, the animals of the CCM ET group, as well as the animals of the WPC + CCM ET group, showed higher levels of NO when compared to the animals of the AIN-93M ET group (*p* < 0.05) (Figure 2f). The groups showed a CV range of 8.5 to 21.8% (Table 3).

### 3.2. Gene Expression of TNF-α, IL-6, and IL-10

**Figure 3:**
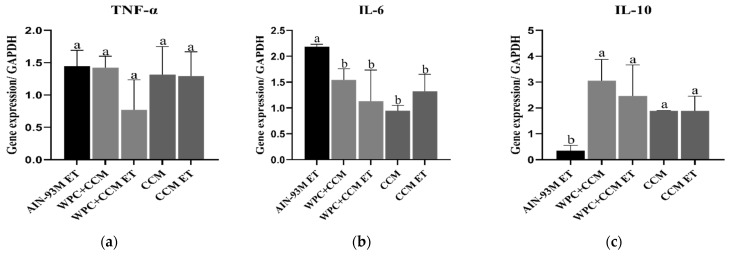
Gene expression of TNF-α, IL-6, and IL-10 in muscle. AIN-93M: group not submitted to ET that received a standard diet was used as the reference group, and the results expressed concerning it; AIN-93M ET: group submitted to the exhaustion test that received a standard diet; WPC + CCM: group not submitted to ET that received curcumin-added whey protein concentrate; WPC + CCM ET: group submitted to the exhaustion test that received curcumin-added whey protein concentrate; CCM: group not submitted to ET that received curcumin; CCM ET: group submitted to the exhaustion test that received curcumin; ET: exhaustion test. The graphs show (**a**) TNF-α, (**b**) IL-6, (**c**) IL-10. According to ANOVA, different lowercase letters (**a**,**b**) indicate significant mean differences among the groups, followed by the Newman–Keuls test, at 5% probability. Same lowercase letters indicate that there was no significant mean difference among the groups. Data expressed as mean ± standard deviation.

The TNF-α expression levels remained similar among groups (Figure 3a). The groups showed a CV range of 12.3 to 58.8% (Table 3). The gene expression of the cytokine IL-6 was higher in the AIN-93M ET group when compared to the other groups (*p* < 0.05) (Figure 3b). The CV range was from 2.4 to 69% (Table 3). The gene expression of the cytokine IL-10 was higher in the groups that received WPC + CCM and CCM, regardless of whether ET was performed, when compared to the AIN-93M ET group (*p* < 0.05) (Figure 3c). The CV range was from 0.3 to 75.7% (Table 3).

### 3.3. Cytokine Production Profile in Muscle Tissue

**Figure 4:**
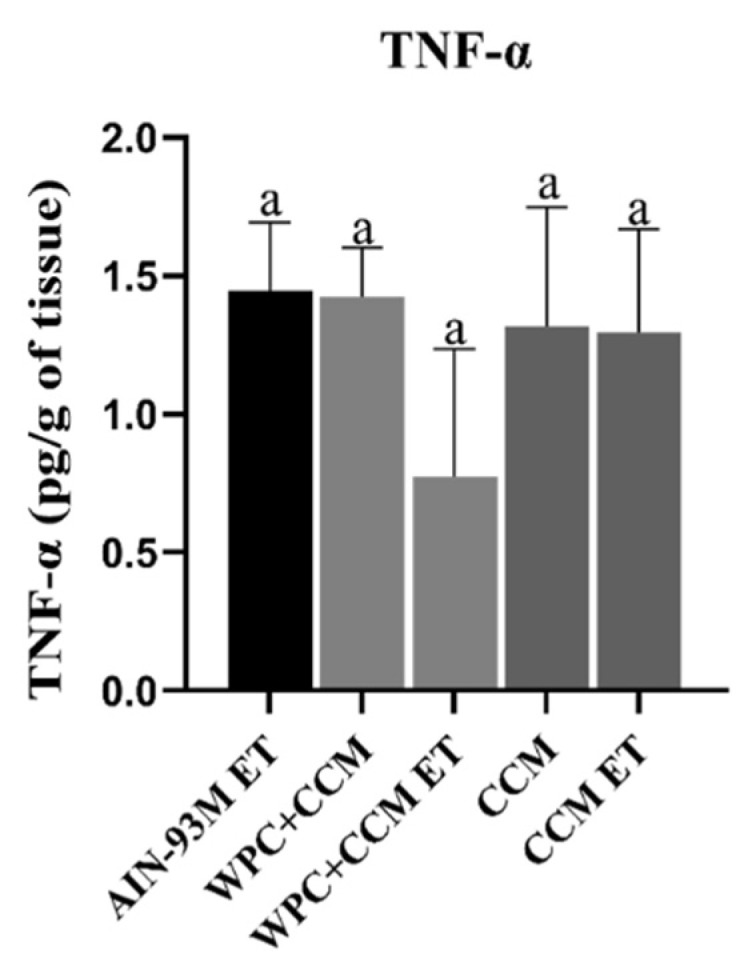
TNF-α profile in muscle using the cytometric bead array kit. AIN-93M: group not submitted to ET that received a standard diet; AIN-93M ET: group submitted to the exhaustion test that received a standard diet; WPC + CCM: group not submitted to ET that received curcumin-added whey protein concentrate; WPC + CCM ET: group submitted to the exhaustion test that received curcumin-added whey protein concentrate; CCM: group not submitted to ET that received curcumin; CCM ET: group submitted to the exhaustion test that received curcumin; ET: exhaustion test. According to ANOVA, different lowercase letters indicate significant mean differences among the groups, followed by the Newman–Keuls test, at 5% probability. Same lowercase letters indicate that there was no significant mean difference among the groups. Data expressed as mean ± standard deviation.

The cytometry-measured levels of cytokines IL-2, IL-10, and TNF-α were calculated in muscle. IL-2 and IL-10 were not detected, probably because they were not yet in their protein form or were below the detection limit.The TNF-α levels among animals not submitted to ET were similar among groups. The animals in the WPC + CCM and CCM group showed a tendency towards a reduction in TNF-α levels compared to the AIN-93M group, but without statistical significance. Among the animals submitted to ET, a trend in the reduction in TNF-α was also observed in the animals of the WPC + CCM ET group (130.8 ± 26.08) when compared to the AIN-93 ET group (196.0 ± 18, 16) and CCM ET (Figure 4). The groups showed a CV range of 8.8 to 48.6% (Table 3).

### 3.4. Histopathology

**Figure 5:**
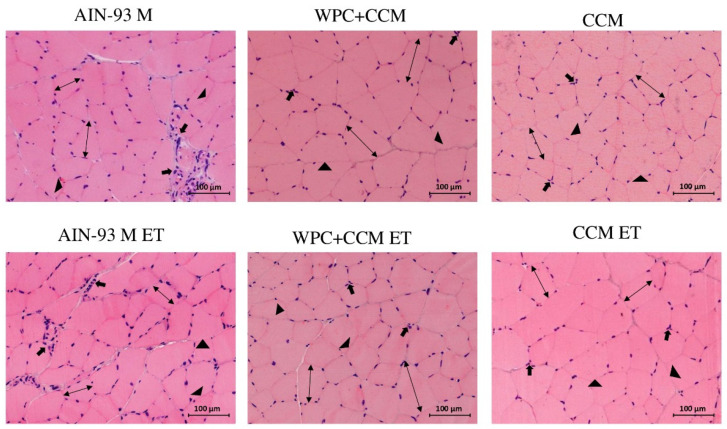
Cross-section of the muscle of rats that were or were not submitted to the exhaustive test. AIN-93M: group not submitted to ET that received a standard diet for rodents in the maintenance phase; AIN-93M ET: group submitted to the exhaustion test that received a standard diet for rodents in the maintenance phase; WPC + CCM: group not submitted to ET that received curcumin-added whey protein concentrate; WPC + CCM ET: group submitted to the exhaustion test that received curcumin-added whey protein concentrate; CCM: group not submitted to ET that received curcumin; CCM ET: group submitted to the exhaustion test that received curcumin. Inflammatory infiltrates (→); Muscle fiber (↔); Connective tissue (►).

Among the animals not submitted to ET, more significant areas of inflammation were observed in the animals of the AIN-93M group (*p* < 0.05) when compared to the animals of the WPC + CCM and CCM groups (Figure 5). The same was observed among the groups submitted to TE, with the WPC + CCM ET and CCM ET groups showing lower inflammation when compared to the AIN-93M ET group (*p* < 0.05) (Table 4). The groups showed a CV range of 8.1 to 28.3% (Table 3).

Among the animals not submitted to ET, it was possible to observe a greater amount of muscle fiber in the WPC + CCM and CCM groups when compared to the AIN-93M group (*p* < 0.05). Among the animals submitted to ET, those in the WPC + CCM and CCM groups had a greater amount of muscle fiber per evaluated area when compared to the animals in the AIN-93M group (*p* < 0.05) (Figure 5). The groups showed a CV range of 1.8 to 2.4% (Table 3). In turn, the presence of connective tissue among the animals not submitted to ET was higher in the AIN-93M group when compared to the WPC + CCM and CCM groups (*p* < 0.05). The same was found among the animals submitted to the exhaustive test, with a greater presence of connective tissue in the muscle tissues of the animals of the AIN-93M ET group when compared to the animals of the WPC + CCM ET and CCM ET groups (*p* < 0.05) (Table 3). The groups showed a CV range of 7.8 to 18.3% (Table 3).

**Figure 6:**
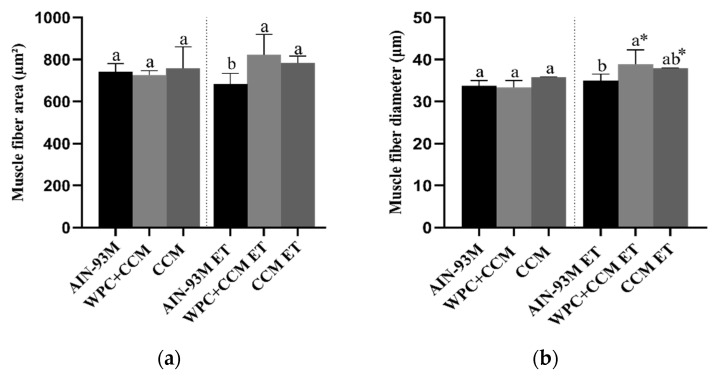
Histopathological analysis of muscle tissue. AIN-93M: group not submitted to ET that received a standard diet; AIN-93M ET: group submitted to the exhaustion test that received a standard diet; WPC + CCM: group not submitted to ET that received curcumin-added whey protein concentrate; WPC + CCM ET: group submitted to the exhaustion test that received curcumin-added whey protein concentrate; CCM: group not submitted to ET that received curcumin; CCM ET: group submitted to the exhaustion test that received curcumin; ET: exhaustion test. The graphs show (**a**) area of muscle fibers (μm²), (**b**) diameter of muscle fibers (μm). * indicates significant differences within the same group (not submitted to ET × submitted to ET), according to the *t*-test (*p* < 0.05). According to ANOVA, different lowercase letters (**a**,**b**) indicate significant mean differences among the groups, followed by the Newman–Keuls test, at 5% probability. Same lowercase letters indicate that there was no significant mean difference among the groups. Data expressed as mean ± standard deviation.

Among the animals not submitted to ET, the area measurements remained similar. Moreover, no differences were observed within the same group (sedentary vs. ET). Among the animals submitted to ET, the animals of the WPC + CCM ET and CCM ET groups had a greater area of muscle fiber when compared to the animals of the AIN-93M ET group, who had muscle atrophy (*p* < 0.05) (Figure 6a). The groups showed a CV range of 2.7 to 14.4% (Table 3).

Regarding the diameter of the muscle fibers, it was possible to observe a greater diameter among the animals in the WPC + CCM ET group when compared to the AIN-93M ET group (*p* < 0.05) (Figure 6b). Among the animals in the CCM ET group, the diameter of the muscle fibers remained similar to those in the WPC + CCM ET and AIN-93M ET groups. The groups showed a CV range of 0.1 to 9.2% (Table 3). Moreover, when comparing the diameters within the same group (not submitted to ET × submitted to ET), the animals of the WPC + CCM ET and CCM ET groups showed greater muscle fiber diameter when compared to the WPC + CCM and CCM groups not submitted to ET, respectively (Figure 6b). No differences were observed among the animals of the AIN-93M group (not submitted to ET × submitted to ET).

### 3.5. Blood Lactate and Swimming Time

**Figure 7:**
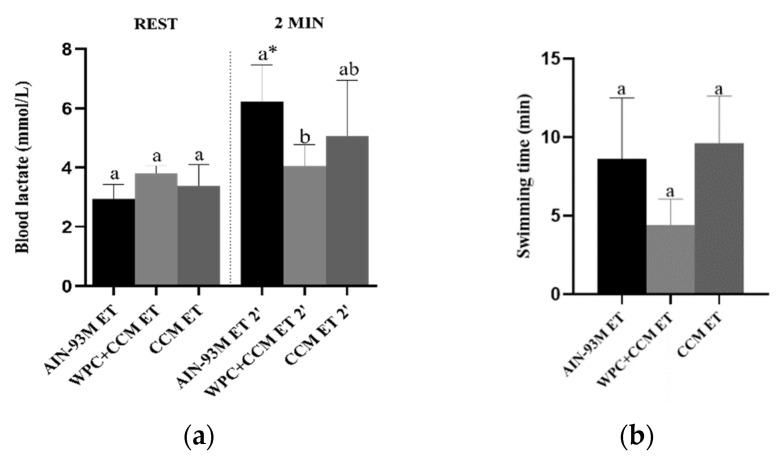
Blood lactate levels and swimming time. (**a**) Blood lactate levels collected at resting and 2 min after performing the exhaustive swimming test. (**b**) Swimming time. AIN-93M: group not submitted to ET that received a standard diet; AIN-93M ET: group submitted to the exhaustion test that received a standard diet; WPC + CCM: group not submitted to ET that received curcumin-added whey protein concentrate; WPC + CCM ET: group submitted to the exhaustion test that received curcumin-added whey protein concentrate; CCM: group not submitted to ET that received curcumin; CCM ET: group submitted to the exhaustion test that received curcumin; ET: exhaustion test. * indicates significant differences within the same treatment group (before ET × after ET), according to the *t*-test (*p* < 0.05). According to ANOVA, different lowercase letters (**a**,**b**) indicate significant mean differences among the groups, followed by the Newman–Keuls test, at 5% probability. Same lowercase letters indicate that there was no significant mean difference among the groups. Data expressed as mean ± standard deviation.

Resting lactate levels and swimming time did not differ among groups. However, after performing the ET, the WPC + CCM group had lower lactate levels when compared to the AIN-93M group (*p* < 0.05). In addition, through comparisons among the groups, it was possible to observe that lactate levels after the end of the ET were higher in the AIN-93M group after the performance of the ET when compared to the same group before ET (*p* < 0.05). In the WPC + CCM and CCM groups, the lactate values before and after performing the ET remained similar (Figure 7). The groups showed a CV range of 16.7 to 41.9% for lactate and 30 to 41.8% for swimming time (Table 3).

## 4. Discussion

Acute and exhaustive exercise is associated with increased tissue stress, especially inflammation, oxidative stress, and, consequently, changes in muscle morphology [2]. In this context, curcumin and whey proteins have been shown to be safe for consumption and exert antioxidant and anti-inflammatory effects [18,21,41]. Overall, our results showed that the consumption of curcumin-added whey protein concentrate had positive effects on oxidative balance and the development of inflammation in the muscle tissue, with a consequent change in muscle morphology. Thus, we can highlight a reduction in oxidative markers, an increase in antioxidant enzymes, and a reduction in inflammation and blood lactate after performing the exhaustive test.

Muscle damage resulting from unusual and exhaustive exercise is related to the production of free radicals and, consequently, ROS associated with inflammation [42], which can lead to cellular damage, such as membrane disorganization, protein oxidation, and the alteration of cellular functions [43]. Increased oxidative stress associated with an inability of the endogenous antioxidant system to remove excess free radicals impairs recovery and decreases exercise performance [42,44].

Substances reactive to thiobarbituric acid (TBARS) or MDA, and carbonyl protein levels are products of the oxidation of biomolecules and are commonly used as biomarkers and indicative of oxidative stress [45]. In turn, enzymes such as CAT, SOD, and GST are used as parameters to reflect the antioxidant status [46]. Our study demonstrated that SOD concentrations remained similar amonggroups, possibly because there was not enough free radical production to activate the antioxidant enzyme system beyond what the cell normally expresses. The curcumin-added whey protein concentrate, as with the exposure to isolated curcumin, provided higher antioxidant activity due to greater recruitment of the catalase enzyme by performing the exhaustive test. Our results show that WPC and CCM can significantly activate antioxidant enzymes in muscle tissue and, consequently, have a protective activity for the tissue. However, surprisingly, CAT concentrations among animals not submitted to ET were lower in the CCM group, probably due to lower H2O2 formation or an ability of curcumin itself to reduce H2O2 levels. It is worth mentioning that our data are based on the analysis of enzyme activity and not on their gene expression; therefore, we believe that future studies are necessary to clarify the possible mechanisms of action and consequent tissue alteration.

The administration of the protein concentrate mixed with curcumin allowed for a reduction in lipid peroxidation induced by free radicals and ROS, such as H_2_O_2_, provoked by the performance of the exhaustive swimming test. Similarly, a study with male rats undergoing resistance exercise training to induce muscle damage also demonstrated the positive modulatory effects of dietary curcumin, with attenuation in MDA concentrations [14]. In the current study, due to its antioxidant properties, CCM was able to reduce protein oxidation, one of the main processes responsible for the misfolding of proteins within the rough endoplasmic reticulum and, consequently, for their malfunction. A previous study by our research group [47] found that CCM consumption was responsible for reducing MDA concentrations, and that exposure to CCM, as well as WPC + CCM, was able to reduce concentrations of PCN in the liver. Our current findings partially corroborate the previous study. The differences found could possibly be related to the investigated tissue, since the liver is the main metabolism organ and, therefore, where the most relevant alterations are found.

Under conditions of oxidative stress, high levels of superoxide ion react with nitric oxide (NO), forming a reactive nitrogen species (RNS), consequently producing peroxynitrite [48]. It is already known that NO suppresses the installation of an inflammatory process by decreasing lymphocyte activation and by decreasing leukocyte adhesion, preventing the rolling and migration of cells and, consequently, inhibiting the installation of a tissue inflammatory process [49,50]. These reports corroborate our findings, since treatments with WPC + CCM and CCM showed increased NO levels and decreased inflammatory infiltrate foci in the tissue.

After unusual and/or prolonged exercise, muscle damage stimulates the inflammatory response through the recruitment of immune system cells, such as neutrophils [42], which send signals for the migration of macrophages that, in turn, mediate the expression of inflammatory cytokines, growth factors, ROS, and RNS directly involved in the progression of inflammation [43]. Thus, the inflammatory process must be controlled to avoid persistent tissue damage due to the continuous action of free radicals [43]. The WPC and CCM represent a promising therapy in controlling inflammation after exhaustive exercise, as reduced IL-6 expression levels and increased IL-10 levels directly reflect attenuation of the inflammatory response provoked by the exercise and improve muscular recovery.

Increased IL-6 in skeletal muscle is related to the incidence of muscle damage induced by strenuous and/or exhaustive exercise [51]. A potential explanation of the lower concentration of IL-6 in the groups that consumed the WPC + CCM or the isolated CCM is probably related to the high concentrations of WPC bioactive peptides and the concentrations of CCM in the blood, which were maintained during and after exercise. Previous studies have also reported that the inflammatory response is suppressed when curcumin is ingested several days before exercise [23,52]. Corroborating our results, Davis et al. (2007) [53] showed that curcumin could reduce IL-6 and TNF-α concentrations after eccentric exercise, controlling inflammation and protecting tissue. We also observed an increase in the levels of IL-10 expression in the muscle of the animals submitted to the exhaustive swimming test, which received the WPC + CCM or the isolated CCM.

IL-10 is an anti-inflammatory cytokine that acts by suppressing the activation of immune cells after the emergence of an initial inflammatory response [54]. The increase in IL-10 concentration can accelerate the inflammatory recovery process [6]. Curcumin has been shown to induce the expression and release of IL-10 [55], which partly explains its anti-inflammatory effects. In our previous study [47], we also observed increased IL-10 concentrations in the liver of Wistar rats that received WPC + CCM or isolated CCM.

The analysis of the inflammatory cytokine profile showed that, although without statistical significance (*p* > 0.05), the WPC + CCM ET group showed a tendency towards lower concentrations of TNF-α. However, levels of IL-10 in its protein form could not be detected. These results suggest that after the production of mRNA for IL-10 (as detected by RT-qPCR analysis), there was a deactivation of M1-type macrophages and an increase in M2-type macrophages reducing exercise-induced inflammation.

The anti-inflammatory role of WPC is related to its concentration of bioactive peptides, present in whey proteins, which can be generated by enzymatic hydrolysis, microbial fermentation, or through the gastrointestinal digestion of food proteins [21]. The ingestion of isolated CCM has been shown to be effective in the reduction in pro-inflammatory cytokines, since CCM can negatively regulate the inducible nitric oxide synthase (iNOS) enzymes, cyclooxygenase-2 (COX-2), lipoxygenase, and xanthine oxidase activity, suppressing the activation of NF-κB [56]. To corroborate our findings, histological evaluations also indicated less inflammation in both the animals that received WPC + CCM and the animals that only received CCM, demonstrating the ability to reduce inflammation. In addition, it was also possible to observe an increase in area and diameter muscle fibers in these groups, which may be related to the increase in muscle strength.

It was also possible to observe that the animals of the control group exhibited a more pronounced connective tissue when compared to the animals of the WPC + CCM and CCM groups. Increased connective tissue, formed mainly by type III collagen fibers, consequently implies decreased muscle fibers. In general, the thickening of collagen fibers is related to some type of injury, usually the beginning of the fibrotic process and cell death [57,58]. These findings are possibly associated with the chronicity of inflammation and the development of degenerative processes that culminate in cell death with consequent collagen deposition. The exhaustive swimming test caused morphological changes that may have led to cell death and increased tissue fibrosis. However, more studies are needed to investigate such effects.

Surprisingly, we observed that the groups that received the AIN93M diet showed increased inflammation in muscle tissue, as well as the development of increased connective tissue and muscle atrophy. The high carbohydrates content and low protein presented in this diet could be related to this result. In its formulation, the AIN93M diet contains 70% of carbohydrates and only 15% of protein impairing protein synthesis. Furthermore, it is known that an increased fats and carbohydrates’ intake in the diet may be related to mechanisms that are involved in lipidic metabolism, such as a reduction in lipoprotein formation, inflammatory processes, and free radicals responsible for lipid chain peroxidation. A previous study by our research group also reported damage caused by the consumption of standard AIN-93M diet by showing that animals fed this diet showed increased fat in hepatocytes.

Exhaustion in swimming rats is a complex type of stress that generates central and peripheral fatigue, since the animals are highly motivated to avoid drowning, and thus, factors such as thermal and emotional stress are difficult to eliminate [30]. In our test of endurance swimming to exhaustion, rats supporting loads of 4% of their body weight usually reached maximal blood lactate stabilization around 4–6 mmol/L, which is indicative of peripheral fatigue [40,59]. More importantly, our results show that the group supplemented with WPC + CCM showed lower lactate levels after the exhaustive swimming test. Elevated levels of lactate in muscle and blood during exercise can provoke sensations of pain and discomfort, increasing the perception of exertion and, consequently, reducing performance [60]. Similar findings were demonstrated by Sahin et al. (2016) [14] in their study with male Wistar rats submitted to an exercise protocol, in which 20 mg of curcuminoids was provided per day for 6 weeks. The authors found that serum lactate levels in the supplemented group decreased compared to control group levels.

This study corroborates our previous findings that the consumption of WPC + CCM and isolated CCM contribute to the reduction in inflammation and oxidative damage caused by the exhaustive swimming test, despite the low dose of CCM offered (3 mg/kg/day). It is important to emphasize that these markers do not directly reflect muscle damage; however, they can be used with other variables to infer muscle changes resulting from exhaustive physical exercise. Future studies are needed to clarify the acute and chronic mechanisms related to the antioxidant and anti-inflammatory responses generated by the consumption of WPC + CCM and isolated CCM, and an evaluation for a longer time after performing an exhaustive test.

## 5. Conclusions

The consumption of WPC + CCM and/or isolated CCM for four weeks before performing an exhaustive swimming test was able to reduce the inflammatory response through the reduction in IL-6 and increase in IL-10. In addition, the consumption of WPC + CCM was able to reduce the lipid peroxidation caused by exhaustive exercise, and the consumption of CCM reduced the concentrations of carbonylated protein. Higher nitric oxide levels were observed by the consumption of WPC + CCM and CCM, evidencing a possible cardioprotective action. Furthermore, the consumption of WPC + CCM and CCM was able to reduce the areas of inflammatory infiltrate and connective tissue in the muscle caused by exhaustive exercise, as well as increase the area and diameter of muscle fibers. The association of WPC + CCM also allowed for lower lactate production during exhaustive exercise.

## Figures and Tables

**Table 1:** Composition of experimental diets.

Ingredients	g/kg of Diet		
	AIN-93M	WPC + CCM	CCM
Cornstarch	455.7	455.7	455.7
Albumin ^1^	150.0	105.0	150.0
Maltodextrin	155.0	155.0	155.0
Sucrose	100.0	99.0	99.0
Soybean oil	40.0	40.0	40.0
Cellulose	50.0	50.0	50.0
Mineral blend	35.0	35.0	35.0
Vitamin blend	10.0	10.0	10.0
L-cystine	1.8	1.8	1.8
Choline bitartrate	2.5	2.5	2.5
WPC ^2^	-	44.0	-
Theracurmin^®^	-	1.2	1.2

Reeves et al., 1993; AIN-93M: standard diet for maintenance phase rodents; WPC: whey protein concentrate; CCM: curcumin. ^1^ Albumin was considered to have 80% protein content. ^2^ WPC with 82.4% protein content was considered.

**Table 2:** Sequences of primers for qPCR analysis.

Genes	Oligonucleotides (5′–3′)	
	Forward	Reverse
GAPDH	CCCCCAATGTATCCGTTGTG	TAGCCCAGGATGCCCTTTAGT
IL-6	TCCTACCCCAACTTCCAATGCTC	TTGGATGGTCTTGGTCCTTAGCC
IL-10	TTGAACCACCCGGCATCTAC	CCAAGGAGTTGTTCCCGTTA
TNF-α	TGGGCTACGGGCTTGTCACTC	GGGGGCCACCACGCTCTT

GAPDH—Glyceraldehyde-3-Phosphate-Dehydrogenase; IL-6—Interleukin-6; IL-10—Interleukin-10; TNF-α—tumor necrosis factor-α.

**Table 3:** Variability expressed as coefficient of variation (CV%), tabulated within different groups.

Biomarkers	CV (%) AIN-93M	CV (%) AIN-93M ET	CV (%) WPC + CCM	CV (%) WPC + CCM ET	CV (%) CCM	CV (%) CCM ET
SOD	2.8	19	2.4	9.4	16.6	15.2
CAT	7.9	15.7	15	4.8	3.5	5
GST	21.2	21.7	15.6	41.5	21.6	15.9
MDA	33.7	18.3	11.2	12.9	22.3	19.5
PCN	14.2	15.8	13.7	23.4	25.5	42.8
NO	11.2	8.5	17.2	21.8	12.1	9.8
IL-6	-	2.4	13.9	69	11.1	26.5
TNF	-	16.2	12.3	58.8	37.9	28.9
IL-10	-	75.7	23.9	54.5	0.3	30.7
TNF (CBA)	48.6	9.2	44.5	18.7	8.8	25.2
Blood lactate	-	20.37	-	16.7	-	41.9
Swimming time	-	37.9	-	41.8	-	30
Histopathology: Inflammation	14.4	22.2	14.6	8.1	14.3	28.3
Point on muscle fiber	2.4	2.3	1.8	1.9	2	1.9
Point on connective tissue	13.6	16.4	7.8	8.2	18.3	9.6
Muscle fiber area	5.1	2.7	14.4	7.1	12.5	4.3
Muscle fiber diameter	3.5	4.8	0.2	4.4	9.2	0.1

AIN-93M: group not submitted to ET that received a standard diet for rodents in the maintenance phase; AIN-93M ET: group submitted to the exhaustion test that received a standard diet for rodents in the maintenance phase; WPC + CCM: group not submitted to ET that received curcumin-added whey protein concentrate; WPC + CCM ET: group submitted to the exhaustion test that received curcumin-added whey protein concentrate; CCM: group not submitted to ET that received curcumin; CCM ET: group submitted to the exhaustion test that received curcumin. CV: coefficient of variation.

**Table 4:** Histopathology of the muscle of rats that were submitted or not to an exhaustive swimming test.

Groups	Inflammation (%)	Count a Point on Muscle Fiber	Count a Point on Connective Tissue
**AIN-93M**	3.69 ± 0.53 ^a^	243.38 ± 5.92 ^b^	43.01 ± 5.88 ^a^
**WPC + CCM**	2.03 ± 0.45 ^b^	254.00 ± 5.72 ^a^	33.70 ± 5.52 ^b^
**CCM**	1.92 ± 0.28 ^b^	258.60 ± 4.59 ^a^	24.60 ± 1.93 ^c^
**AIN-93M ET**	3.59 ± 0.29 ^a^ª	236.80 ± 4.43 ^b^	39.31 ± 3.23 ^a^
**WPC + CCM ET**	2.02 ± 0.29 ^b^	259.15 ± 5.08 ^a^	26.65 ± 4.88 ^b^
**CCM ET**	1.73 ± 0.49 ^b^	258.06 ± 4.88 ^a^	26.46 ± 2.55 ^b^

AIN-93M: group not submitted to ET that received a standard diet; AIN-93M ET: group submitted to the exhaustion test that received a standard diet; WPC + CCM: group not submitted to ET that received curcumin-added whey protein concentrate; WPC + CCM ET: group submitted to the exhaustion test that received curcumin-added whey protein concentrate; CCM: group not submitted to ET that received curcumin; CCM ET: group submitted to the exhaustion test that received curcumin; ET: exhaustion test. Means followed by the same letter in the column do not differ for the same condition (not submitted to ET × submitted to ET). According to ANOVA, different lowercase letters (**a**–**c**) indicate intra-group differences, followed by the Newman–Keuls test, at 5% probability. Same lowercase letters indicate that there was no significant mean difference among the groups. Data expressed as mean ± standard deviation.

## Data Availability

Data can be made available on request.
